# P17 Aetiology of hospital-acquired Gram-negative bacteraemias over the COVID-19 pandemic: a single NHS Trust experience, UK

**DOI:** 10.1093/jacamr/dlad066.021

**Published:** 2023-06-14

**Authors:** Syra Dhillon, Oscar Buchanan, Catherine Sargent

**Affiliations:** Royal Sussex County Hospital, Brighton, UK; Royal Sussex County Hospital, Brighton, UK; Royal Sussex County Hospital, Brighton, UK

## Abstract

**Background:**

Gram-negative bacteria (GNB) are the most common cause of healthcare-associated bacteraemias, most commonly caused by *Escherichia coli*, *Klebsiella*, and *Pseudomonas.*^1^ GNB carry significant mortality and morbidity; therefore, prompt and appropriate antibiotic therapy is important.^2^ However, there are also high rates of GNB resistance, posing a significant public health concern. Nationally, all NHS trusts are mandated to report their rates of GNB to the UK Health Security Agency as part of their plan to reduce incidence by 2025.^3^ The COVID-19 pandemic disrupted healthcare delivery from March 2020, with higher rates of GNB being recorded nationally.^4^

**Methods:**

We retrospectively analysed 3 years of GNB data, using a 5-month sample of April to August each year, from University Sussex Hospital NHS Foundation Trust east hospitals. Looking at hospital-acquired data (defined as blood culture taking on or after ay 3 of admission), we analysed the demographics, incidence and resistance patterns of GNB using online medical and microbiology records. Data collection and analysis took place on Microsoft Excel.

**Results:**

From 2019 to 2021, there were 29, 27 and 19 cases in each time 5-month sample, respectively. The median age of patients was 78, 64 and 73 years. Positive blood cultures were taken later into admission during 2020 (14 versus 8 and 9 days) with an increased length of median hospital stay (33 versus 23 and 24 days). The source of each GNB can be seen in Table 1 (created as per national record keeping guidelines), with 2020 attributing a higher proportion of cases to ‘other’, which includes iatrogenic procedures and surgical operations. Mortality rates were highest in 2020 (26.9 versus 21.4 and 21.1%), although not significantly, with transmission in neonates a risk factor. 2020 also carried the highest rate of multi-drug–resistant GNB (22%), both within and outside of an intensive care setting.

**Conclusions:**

Our data shows an increase in the mortality rate and antibiotic resistance in GNB during the height of the COVID-19 pandemic that returned to baseline the following year, likely due to a reduction in elective activity and fewer bed days. Ongoing evaluation of GNB aetiology, particularly resistance patterns, is vital to improve health outcomes.

**Table 1.** Incidence and likely source of GNB over a 5-month period across 3 years

Overall GNBs	Gastrointestinal, *n* (%)	Hepatobiliary, *n* (%)	Urine, *n* (%)	Chest, *n* (%)	Other, *n* (%)	Unknown, *n* (%)
2019	3 (10.3)	4 (13.8)	5 (17.2)	7 (24.1)	1 (3.4)	9 (31.0)
2020	1 (3.7)	4 (14.8)	4 (14.8)	1 (3.7)	9 (33.3)	5 (18.5)
2021	0 (0.0)	4 (21.0)	2 (10.5)	2 (10.5)	3 (15.8)	8 (42.1)
